# Supplementary material for: Internal amino acid state modulates yeast taste neurons to support protein homeostasis in Drosophila
Source: eLife. 2018 Feb 2;7:e31625. doi: 10.7554/eLife.31625 (PMC5812714; doi:10.7554/eLife.31625)
Supplement: Supplementary file 1. [file elife-31625-supp1.pdf]

line	genotype	source	yeast preference phenotype	note
Ir25a-GAL4	*w; IR25a-Gal4 #236.1/(CyO);*	BDSC	Yes	
Ir76b-GAL4	*w; IR76B-Gal4 #226.8/CyO;*	BDSC	Yes	
1B-GAL4	*d5-HT 1B-Gal4*	Amita Sehgal	No	
ato-GAL4	*y[1] w[*]; P{w[+mC]=ato-GAL4.3.6}10;*	BDSC	No	
ato-GAL4	*y[1] w[*]; P{w[+mC]=ato-GAL4.3.6}14a;*	BDSC	No	
c316-GAL4	*w[*];; P{w[+mW.hs]=GawB}c316*	BDSC	Yes	also expresses in taste pegs and other gustatory neurons
DCGLUT-GAL4	*DCGLUT-Gal4*	Aaron DiAntonio	Yes	severe secondary behavioral impairments
Ddc-GAL4	*w[1118]; P{w[+mC]=Ddc-GAL4.L}Lmpt[4.36];*	BDSC	No	
E409-GAL4	*w[*]; P{w[+mW.hs]=GawB}E409;*	BDSC	No	
ehups-GAL4	*w*; P[w+ Ehups-Gal4]C21;*	Elizabeth Gavis	No	
G0338-GAL4	*P{w[+mGT]=GT1}CG4004 [BG01793] w[1118];;*	BDSC	No	
G0431-GAL4		Ann-Shyn Chiang	No	
Gr2a-GAL4	*w[*]; P{w[+mC]=Gr2a-GAL4.2.2}3;*	BDSC	No	
Gr22c-GAL4	*w*; P{Gr22c-GAL4.D}2/CyO;*	BDSC	No	
Gr23a-GAL4 (III)	*w*;; P{Gr23a-GAL4.3.5}1*	BDSC	No	
Gr23a-GAL4 (II)	*w*; P{Gr23a-GAL4.3.5}5/CyO;*	BDSC	No	
Gr28a-GAL4	*P{w[+mC]=Gr28a-GAL4.T}E1/TM3, Sb[1]*	BDSC	No	
Gr39a.b-GAL4	*;;P{w[+mC]=Gr39a.b-GAL4.1}1D*	BDSC	No	
Gr57a-GAL4	*w[*]; P{w[+mC]=Gr57a-GAL4.6}1D/CyO*	BDSC	No	
Gr5a-GAL4	*;Gr5a-Gal4; Gr5a-Gal4*	Kristin Scott	No	
Gr64e-GAL4	*w[*]; P{w[+mC]=Gr64e-GAL4.8}5;*	BDSC	No	

Gr77a-GAL4 (III)	*w*;; P{Gr77a-GAL4.4}1L*	BDSC	No	
Gr77a-GAL4 (II)	*w*; P{Gr77a-GAL4.4}10;*	BDSC	No	
Gr89a-GAL4	*w*; P{Gr89a-GAL4.2}11/CyO*	BDSC	No	
Gr92a-GAL4 (II)	*w*; P{Gr92a-GAL4.3.9}6;*	BDSC	No	
Gr92a-GAL4 (III)	*w*;; P{Gr92a-GAL4.3.9}1/TM3, Sb1*	BDSC	No	
Gr94a-GAL4	*w[*]; P{w[+mC]=Gr94a-GAL4.3.7}10;*	BDSC	No	
Gr98c-GAL4	*w*; P{Gr98c-GAL4.4}3/CyO;*	BDSC	No	
Gr94a-GAL4	*w*;; P{Gr94a-GAL4.3.7}6*	BDSC	No	
Gr9a-GAL4 (II)	*w*; P{Gr9a-GAL4.6.3}7;*	BDSC	No	
Gr9a-GAL4 (III)	*w*;; P{Gr9a-GAL4.6.3}1*	BDSC	No	
HugS3-GAL4	*w[*]; P{w[+mC]=Hug-GAL4.S3}3*	Michael Pankratz	No	
Ilp7-GAL4	*W* (I);; ILp7-Gal4/(TM6SbDfdYFP)*	Irene Miguel-Aliaga	No	
Ir20a-GAL4	*w[*];; P{w[+mC]=Ir20a-GAL4.K}2/TM6B, Tb[1]*	BDSC	No	
Ir40a-GAL4	*w; IR40a-Gal4 #214.1/(CyO);*	Richard Benton	No	
Ir41a-GAL4	*w; IR41a-Gal4 BT42.1/CyO;*	Richard Benton	No	
Ir52d-GAL4	*w[*];; P{y[+t7.7] w[+mC]=Ir52d-GAL4.K}attP2/TM6B, Tb[1]*	BDSC	No	
Ir56a-GAL4	*w[*];; P{w[+mC]=Ir56a-GAL4.K}3*	BDSC	No	
Ir56b-GAL4	*w[*]; P{y[+t7.7] w[+mC]=Ir56b-GAL4.K}attP40/CyO;*	BDSC	No	
Ir56d-GAL4 (III)	*; IR56d-Gal4 BT76.1 #3/TM6b*	Richard Benton	No	
Ir56d-GAL4 (II)	*w*; P{Ir56d-GAL4.K}7-2/CyO;*	BDSC	No	
Ir60b-GAL4	*w[*]; P{y[+t7.7] w[+mC]=Ir60b-GAL4.K}attP40/CyO;*	BDSC	No	

Ir67c-GAL4	*w[*]; P{y[+t7.7] w[+mC]=Ir67c-GAL4.Kb}attP40/CyO;*	BDSC	No	
Ir75a-GAL4	*w[*];; P{w[+mC]=Ir75a-GAL4.S}BT12.1/TM6B, Tb[1]*	BDSC	No	
Ir75a-GAL4	*w;; IR75a (5’+3’)-Gal4 BT12.1*	Richard Benton	No	
Ir76a-GAL4	*w; IR76a-Gal4 #292.3B/CyO;*	Richard Benton	No	
Ir8a-GAL4	*w; IR8a-Gal4 #204.8/CyO;*	Richard Benton	No	
Ir92a-GAL4	*w;; IR92a-Gal4 #288.3/TM2*	Richard Benton	No	
Ir94a-GAL4	*w[*];; P{w[+mC]=Ir94a-GAL4.K}4*	BDSC	No	
Ir94c-GAL4	*w[*]; P{y[+t7.7] w[+mC]=Ir94c-GAL4.Kb}attP40/CyO;*	BDSC	No	
Ir94f-GAL4	*w[*]; P{y[+t7.7] w[+mC]=Ir94f-GAL4.K}attP40/CyO;*	BDSC	No	
Ir94h-GAL4	*w[*]; P{y[+t7.7] w[+mC]=Ir94h-GAL4.K}attP40/CyO;*	BDSC	No	
LEUC-GAL4	*;LEUC-GAL4/Cyo;*	Bader Al-Anzi	No	
npf-GAL4	*;npf-Gal4;*	Ping Shen	No	
Poxn-GAL4 (II)	*;Poxn-Gal4 T2d;*	Ribeiro lab	No	
Poxn-GAL4 (III)	*;;Poxn-Gal4 T2a/TM3,Sb*	Ribeiro lab	No	
Tdc2-GAL4	*w[*]; P{w[+mC]=Tdc2-GAL4.C}2;*	BDSC	No	
TH-GAL4	*w*(I);; P{ple-GAL4.F}3*	BDSC	No	
VT30559-GAL4	*P{VT030559-GAL4}attP2*	VDRC	No	
VT61723-GAL4	*P{VT061723-GAL4}attP2*	VDRC	No	

Supplementary Table 1: GAL4 lines screened for yeast preference phenotypes using thermogenetic silencing.

Line	LSO (cluster 1)	LSO (cluster 2)	DCSO	VCSO
1261-GAL4;UAS-CD8::GFP	2	0	2	3
1261-GAL4;Poxn-GAL80;UAS-CD8::GFP	0	0	0	0
Ir25a-GAL4;UAS-CD8::GFP	6	2	6	3
Ir25a-GAL4; Poxn-GAL80;UAS-CD8::GFP	0	0	0	0
Ir76b-GAL4;UAS-CD8::GFP	6	2	6	3
Ir76b-GAL4; Poxn-GAL80;UAS-CD8::GFP	3	0	0	0

Supplementary Table 2: Number of cell bodies counted on either side of the internal sense organs in the depicted lines.

figure	line	full genotype	source
1,2,3,4,5,6,1S1,1S2,1S3,3S3,4S1,5S1,6S1	*;Ir76b-GAL4;*	*w; IR76B-Gal4 #226.8/CyO;*	Richard Benton
1,2,S1,1S2,1S3,2S1	*;Ir25a-GAL4;*	*w; IR25a-Gal4 #236.1/(CyO);*	Richard Benton
1,2,3,S1,1S2,1S3,2S1,3S3	*;1261-GAL4;*	*w*;1261-GAL4;(CyO)*	Ulrike Heberlein
1,2,1S2,2S1,2S2	*;UAS-CD8::GFP;*	*;UAS-CD8::GFP/(CyO);*	Barry Dickson
1,2,1S2,2S1	*;UAS-shi[ts] (attP5);*	*pJFRC100-20xUAS-TTS-Shibire-ts1-p10 in su(Hw) attP5*	Gerry Rubin
1,2,1S2,2S1	*;attP5; control*	*y[1] w[*]; P{y[+t7.7]=CaryIP}su(Hw)attP5*	BDSC
1,3,4,5,1S1,1S3,4S1,5S1	*;UAS-GCaMP6s;*	*w[1118]; P{y[+t7.7] w[+mC]=20XUAS-IVS-GCaMP6s}attP40*	BDSC
1,1S3	*;;Ir76b-GAL80 (attP2)*		Ribeiro lab, see methods
2	*;tshirt-GAL80;*		Gero Miesenboeck
2,3	*;Poxn[ΔM22-B5];*		Werner Boll
2,3	*;Poxn[ΔM22-B5];ΔSfoBs105/ΔSfoBs127*		Werner Boll
2	*;;VT033654-GAL4 (attP2)*		VDRC
2	*;Gr9a-GAL4;*	*w*; P{Gr9a-GAL4.6.3}7;*	BDSC
2	*;Ir67c-GAL4;*	*w[*]; P{y[+t7.7] w[+mC]=Ir67c-GAL4.Kb}attP40/CyO;*	BDSC
2,3,1S1,1S2,1S3,2S1,2S2,3S3	*;;UAS-shi[ts] (VK00005)*	*pJFRC100-20xUAS-TTS-Shibire-ts1-p10 in VK00005*	Janelia Research Campus
2,3,1S1,1S2,1S3,2S1,2S2,3S3	*;;VK00005 control*	*y[1] w[1118];; PBac{y[+]-attP-9A}VK00005*	BDSC
2,2S1	*;;Poxn-GAL80 (attP2)*		Ribeiro lab, see methods
2,3,2S2,3S3	*;;67E03-GAL4*	*w1118;; P{GMR67E03-GAL4}attP2*	BDSC
2,3,2S2,3S3	*;;pBDP-GAL4U (attP2)*		BDSC
3, 2S2,3S3	*;;57F03-Gal4*	*w1118;; P{GMR57F03-GAL4}attP2*	BDSC
3,3S3	*attp2*	*y[1] w[67c23];;P{CaryP}attP2*	BDSC
3,3S3	*; ;UAS-GtACR1*	*w[1118];; 20xUAS-GtACR1 (attP2)*	Adam Claridge-Chang

3,2S2	*;;Poxn-GAL4*	*w*;P{Poxn-Gal4.14}1-7/TM6B, Tb*[1]	Markus Noll
3	*;UAS-Kir2.1;*	*w*;P{UAS-Hsap\KCNJ2.EGFP}1;*	BDSC
4,2S2,3S3,5S1	*;Gr5a-GAL4;*		Kristin Scott
1S1,3S3	*E409-GAL4*	*w[*]; P{w[+mW.hs]=GawB}E409;*	BDSC
1S1	*Gr64e-GAL4*	*w[*]; P{w[+mC]=Gr64e-GAL4.8}5;*	BDSC
1S2,3S3	*w[1118];;*		Barry Dickson
1S3	*Ir76b[1]*		Craig Montell
1S3	*Ir76b[rev]*		Craig Montell
1S3	*Ir76b[MB00216]*	*y[1]w[67c23];;Mi{ET1}Ir76b[MB00216]*	BDSC
1S3	*Ir76b[05]*	*y[1]w[*];;P{UAST-YFP.Rab39.S23N}Ir76b[05]*	BDSC
1S3	*Ir25a[1]*		Richard Benton
1S3	*IR25a[2]*		Richard Benton
2S1	*Ir8a[-/-]*	*Ir8a[1];;*	Richard Benton
2S1	*Ir8a[+/+]*	*IR8a[1], IR8a-rescue # BT128.1;;*	Richard Benton
2S1	*Orco[1]*		Sofia Lavista-Llanos
2S1	*Orco[2]*		Sofia Lavista-Llanos
2S1	*;;Orco-GAL4*	*w[1118]; P{w[+mC]=Orco-GAL4.K}97.1*	BDSC
2S1	*;Ir8a-GAL4;*	*w; IR8a-Gal4 #204.8/CyO;*	Richard Benton
2S1	*atonal[-/-]*[-]	*eyflp; FRT82B ato[1]/FRT82B, CL*	Ilona Kadow
2S1	*atonal[+/+]*	*eyflp; FRT82B/FRT82B, CL*	Ilona Kadow
2S3	*;Ir60b-GAL4;*	*w; IR60b-Gal4[B-P40-1]/CyO;*	BDSC
2S3	*;;Ir94f-GAL4*	*P{y[+t7.7] w[+mC]=Ir94f-GAL4.K}attP2*	BDSC
2S3	*;Ir94a-GAL4;*	*w[*]; P{w[+mC]=Ir94a-GAL4.K}5/CyO;*	BDSC
2S3	*;Gr2a-GAL4;*	*w[*]; P{w[+mC]=Gr2a-GAL4.2.2}3;*	BDSC

2S3	*;Gr23a-GAL4;*	*w*; P{Gr23a-GAL4.3.5}5/CyO;*	BDSC
2S3	*;Gr77a-GAL4;*	*w*; P{Gr77a-GAL4.4}10;*	BDSC
2S3	*;Gr92a-GAL4;*	*w*; P{Gr92a-GAL4.3.9}6;*	BDSC
2S3	*;Gr22c-GAL4;*	*w*; P{Gr22c-GAL4.D}2/CyO;*	BDSC
2S3	*;;Gr39a.b-GAL4*	*P{w[+mC]=Gr39a.b-GAL4.1}1D*	BDSC
2S3	*;Gr98c-GAL4;*	*w*; P{Gr98c-GAL4.4}3/CyO;*	BDSC
2S3	*;;Gr28a-GAL4*	*P{w[+mC]=Gr28a-GAL4.T}E1/TM3, Sb[1]*	BDSC
2S3	*;Gr57a-GAL4;*	*w[*]; P{w[+mC]=Gr57a-GAL4.6}1D/CyO*	BDSC
2S3	*;Gr89a-GAL4;*	*w*; P{Gr89a-GAL4.2}11/CyO*	BDSC
3S3	*83F01-Gal4*	*w[1118];; P{GMR83F01-GAL4}attP2*	BDSC
6S1	*UAS-SPR-IR1*		Barry Dickson
6S1	*;UAS-SPR-IR2;*		Barry Dickson
6S1	*UAS-Dcr2;;*	*w[1118], P{w[+mC]=UAS-Dcr-2};;*	Barry Dickson

Supplementary Table 3: Genetic lines used in the manuscript.
